# Spanish Version of the Everyday Discrimination Scale (EDS-E): Factorial Structure and Scale Invariance in Spanish Adolescents

**DOI:** 10.3390/jcm14092887

**Published:** 2025-04-22

**Authors:** Alejandro Miguel-Alvaro, Jairo Rodríguez-Medina, Clara González-Sanguino

**Affiliations:** 1 Department of Psychology, Faculty of Law, University of Valladolid, C/Plaza de Santa Cruz, 8, 47002 Valladolid, Spain; 2 Department of Pedagogy, Faculty of Education and Social Work, University of Valladolid, C/Plaza de Santa Cruz, 8, 47002 Valladolid, Spain; jairo.rodriguez.medina@uva.es; 3 Department of Psychology, Faculty of Education and Social Work, University of Valladolid, C/Plaza de Santa Cruz, 8, 47002 Valladolid, Spain; clara.gonzalez.sanguino@uva.es

**Keywords:** EDS, perceived discrimination, adolescence

## Abstract

**Background/Objectives:** Discrimination is a phenomenon of special relevance in adolescence, as this is a key period in the development of young people, so measures that accurately and reliably assess it are essential. The aim of this research is to study the psychometric properties of the Spanish version of the Everyday Discrimination Scale in a sample of Spanish adolescents. **Methods:** The scale was applied to 1000 adolescents using Computer Assistance Web Interview (CAWI) methodology by means of a stratified random sampling by age, gender and territorial distribution. **Results:** The results reveal an unifactorial structure of the scale, with adequate measures of reliability and validity that confirm that it is a suitable instrument for assessing everyday discrimination in this population. **Conclusions**: This study has implications for understanding the experiences of discrimination in adolescents and for developing interventions to reduce discrimination and promote equality. Limitations and implications for the future are also discussed.

## 1. Introduction

Discrimination involves treating individuals or groups differently based on certain characteristics, which can be either relevant or irrelevant to the context. When these characteristics are irrelevant, such as race, gender or sexual orientation, the treatment is often considered unfair and potentially illegal [1]. Discrimination can take various forms, including direct and indirect discrimination, harassment and victimization, and can originate from multiple sources like peers, parents, educators and healthcare professionals [2,3]. It can be based on race, gender, age, disability, sexual orientation and other characteristics [4], and it is widely studied that discrimination affects people’s social and economic status, well-being and health [5]. It also has broader social implications, contributing to systemic inequalities and social injustice. Specifically, as highlighted in works such as that of Trent et al. [6], any form of discrimination can leave damage, sometimes long-lasting and with dramatic effects such as low self-esteem, deep feelings of loneliness, exclusion, marginalization, isolation, a parasitic lifestyle with little social involvement, poor interpersonal relationships and little or no motivation for anything that represents the environment in which marginalization occurs (e.g., school), and others [6].

Adolescence is a critical developmental period characterized by significant physical, social and emotional changes. During this time, individuals are particularly vulnerable to the adverse effects of discrimination, which can have profound implications on their overall development and well-being [7]. Specifically, discrimination in the adolescent period is associated with an increased risk of depression, anxiety and other psychological problems [8]. Adolescents who experience discrimination tend to have higher levels of stress and psychological maladjustment [7]. In addition, studies indicate that the stress associated with discrimination can lead to behavioral health problems such as substance use and attention deficit hyperactivity disorder [9]. On the other hand, discrimination in the school environment can have a negative impact on academic performance and engagement. Adolescents who perceive a negative racial/ethnic climate in their schools are more likely to experience discriminatory treatment, which in turn affects their academic outcomes [10]. Discrimination can hinder the process of social identity development in adolescence, leading to self-esteem problems [11]. Experiences of discrimination can also affect adolescents’ relationships with parents and peers, which can lead to social isolation and reduced support systems [12]. Taking all the above into consideration, it is clear how important it is to understand and address discrimination during adolescence because of its far-reaching effects on mental health, academic performance and social development.

Specifically, discrimination among adolescents in Spain is multifaceted and affects various groups differently depending on their physical appearance [3], ethnicity [13], gender [14] and sexual orientation [15]. The most recent study with Spanish adolescents revealed that 66% of them reported having witnessed or suffered discrimination, mainly in the form of teasing, insults and bullying, with peers being the main perpetrators [3]. In Spain, schools [3] and social networks [14], are primary contexts for these behaviors, which require specific and early interventions to address and mitigate the impact on adolescents’ well-being and mental health. However, in order to do so, a first aspect of vital importance is to have quality psychometric instruments that allow for the detection and evaluation of such discrimination in adolescence.

There are many scales that seek to measure discrimination. Among them, we can highlight the Racism and Life Experiences Scales [16] and the Experience of Discrimination Scale (EOD) [17], which measures the frequency with which people are discriminated against based on their race; the Discrimination and Stigma Scale (DISC-12) designed to assess the extent and content of discrimination in people with mental health problems [18]; the Religious Discrimination Scale (RDS), which assesses aspects of discrimination applicable and generalizable to various religious affiliations [19]; and the Everyday Discrimination Scale (EDS) developed by Williams et al. [20], which has been used to measure perceived discrimination on a day-to-day basis. With the child and youth population, there are specific scales such as the Adolescent Discrimination Distress Index [21]; the Perceived Discrimination Scale for Chinese Migrant Adolescents [22]; Perceived Racial Discrimination at School [23]; and the aforementioned EDS [20].

Precisely, of all the measures proposed to measure discrimination, the Everyday Discrimination Scale (EDS) is one of the most widely used instruments in different populations [24,25]. Although the EDS was originally developed with a focus on racial mistreatment of ethnic minorities, researchers regularly use it to assess other types of mistreatment, such as discrimination based on mental health conditions, gender or body weight [26,27]. The EDS was developed by Williams et al. [20] to assess the frequency of discrimination experiences in people’s daily lives. These experiences include being treated with less courtesy and respect than other people; receiving worse service in restaurants or stores; having people act as if one is inferior, frightening, dishonest or unintelligent; being called names; or receiving threats or harassment.

Empirical evidence indicates that the different versions exhibit quality psychometric properties [28]. The EDS has demonstrated high internal consistency across various studies [24]. For example, in a study with Black adolescents, the alpha reliability coefficient was 0.87, and the split-half reliability was 0.83 [29]. Another study reported a Cronbach’s alpha of 0.89 for a modified version of the EDS adapted for medical settings [30]. Furthermore, the EDS has shown adequate construct validity [31]. Additionally, the EDS scores were significantly related to internalizing and externalizing symptoms, indicating good convergent validity [29]. The EDS is generally considered unidimensional, although some studies have identified local dependence or nuisance multidimensionality. For example, an exploratory factor analysis with African American law students indicated local dependence within the EDS [31]. In addition to such good general psychometric properties, the EDS has other strengths. Among them, it can be highlighted that it considers different reasons of discrimination and different contexts, and has a holistic approach, including not only macroaggressions, but also microaggressions [20].

Several validations of the EDS have been conducted with samples of adolescents. For instance, Black American adolescents [29] or Portuguese adolescents [32]. However, we do not currently have a study in which EDS has been tested in a sample of Spanish adolescents. In fact, there are validation studies with the EDS in Spanish, but not with a Spanish sample, since they have been carried out with Colombian adults [33] or with Chilean children and adolescents [34]. As mentioned above, the factor structure of the EDS in adolescents shows significant variations in different studies, indicating discrepancies in its application and validation in this population. The factor structure may vary according to cultural context and demographic group. For example, the Portuguese adaptation of the EDS for adolescents [32] showed a two-factor structure, while studies in Black adolescents in the USA found only one factor [29]. The validity and reliability of the EDS may depend on how it is adapted and validated in different adolescent populations. The Portuguese adaptation showed that the EDS can distinguish between perceptions of discrimination in different groups, suggesting that it may be a useful tool for measuring discrimination in specific contexts [32]. Therefore, although the psychometric properties of the EDS have been studied in the Spanish version [33,34], it is crucial to consider the cultural and demographic context when using the EDS with adolescents. These studies were conducted with a Latin American, not a Spanish, population. The variability in the factor structure suggests that specific adaptations and validations should be carried out to ensure the accuracy and relevance of the scale in different groups [35].

Moreover, if we take into consideration that some authors claim that it is necessary to update the theoretical foundations of the EDS to ensure its relevance in diverse populations [36], studying the psychometric properties of the EDS in a population in which this has not yet been done (Spanish adolescents) is highly relevant. Even more so, taking into account the magnitude of the problem through the data expressed previously in which 66% of Spanish adolescents declared having witnessed or suffered discrimination, mainly in the form of mockery, insults and bullying, with their peers being the main aggressors and the school being the main context [3].

Based on the above, the aim of the present study becomes relevant, namely to analyze the psychometric properties of the EDS in a Spanish sample of adolescents in order to explore whether this scale is an adequate instrument to assess discrimination in this specific sample.

## 2. Methods

### 2.1. Procedure

This study is part of a wider research project “Stigma and discrimination as factors of vulnerability in childhood” funded by La Caixa Foundation (FS23-1B073). The study design is quantitative, exploratory, descriptive and cross-sectional. We used stratified random sampling according to age, gender and territorial distribution through a panel of families, with data collection between October and November 2023. Participants met the following inclusion criteria: (a) aged 12–16 years, (b) access to the internet and a mobile device or computer, and those who did not have an adequate level of Spanish to complete the instrument were excluded.

The company “Análisis e Investigación” managed the sampling and data collection under CCI/Esomar ethical standards and ISO 20,252 [37] and ISO 27,001 [38] certifications, ensuring the quality of the process. Data were collected through an online survey using CAWI (Computer-Assisted Web Interview) methodology and a certified “access panel” with more than 100,000 panelists. Recruitment of informants was conducted by invitation through personalized links to parents or legal guardians. Information about the study was provided and informed consent was obtained before adolescents responded to the surveys autonomously via devices such as PC, tablet or smartphone.

A cross-sectional natural group design (NGD) was used to assess the consistency of the responses of the adolescents and their guardians to the items of the instrument. The questionnaire, divided into two parts, included a socio-demographic section for adults (average length: 5 min) and a longer section for adolescents (average length: 25 min), where several scales were included. All data collected were anonymous, and the study was approved by the Ethics Commission of the University of Valladolid (protocol code PI 23-3245NOHCUV).

### 2.2. Participants

A total of 1000 Spanish adolescents participated by means of stratified random sampling in terms of age, gender and territorial distribution (sampling error 3.1% with a confidence level of 95.5% for an infinite universe and under the assumption of maximum indeterminacy). In terms of gender, 50% were female and 49.3% male and 0.7% did not identify with any of the above. The mean age was 14 (SD = 1.41). Most of the families resided in large municipalities (more than 50% live in cities with more than 200,000 inhabitants), as well as having a high level of education (more than 57% have university or postgraduate studies), and an average monthly family income.

The adolescents interviewed mostly attended public schools (69.5%) and, in terms of belonging to ethnic minorities, only 15.5% of the sample considered themselves to belong to an ethnic group other than North European or North American. With regard to the presence of a mental disorder, physical illness or disability, 92.7% of parents indicated that their children did not have or had not had any of these. Details of the sociodemographic characteristics of the sample can be seen in Supplementary Materials (Table S1).

### 2.3. Variables and Instruments

- Socio-demographic characteristics. A series of ad hoc questions are included for the parents of the adolescents: (1) Age of the adolescent; (2) Monthly income; (3) Highest level of education in the household; (4) Highest level of education in the household; (5) Type of educational center attended by the child; (6) If the minor has or has had any mental disorder, physical illness or disability. In the same way, a series of questions designed ad hoc to be answered by minors are included: (1) Gender; (2) Ethnicity.
- Discrimination. The Everyday Discrimination Scale (EDS) [39] was used, in its Spanish version [33,34]. The scale is a 9-item instrument that inquiries about experiences of everyday discrimination, such as “You are treated with less respect than other people” or “People act as if they are better than you.” Items are rated on a Likert scale (0 = never, 1 = almost every day, 5 = less than once a year). Higher scores indicate greater perceived discrimination. Finally, participants are asked to specify the reason(s) for these experiences, including: ancestry or origin, gender, age, religion, height, appearance, sexual orientation, family education level, family income, disability, physical illness, weight or other specified reasons. The obtained scores are transformed to a scale of 0 to 100 points, where higher scores indicate higher levels of perceived discrimination.

### 2.4. Data Analysis

The analysis was conducted in four phases. In the first phase, a descriptive analysis of the entire dataset was performed. In the second phase, after verifying the suitability of the data for factor analysis using the Kaiser–Meyer–Olkin (KMO) test and Bartlett’s sphericity test, an optimized parallel analysis was carried out [40]. Additionally, in this phase, the presence of multivariate outliers was checked using Mahalanobis D^2^ distances and Guttman errors. In Phase 3, to verify the instrument’s unidimensionality, Mokken Scale Analysis (MSA) was used. Item scalability was evaluated using Loevinger’s homogeneity coefficient (H). The obtained homogeneity coefficients (H) allow for the evaluation of the unidimensionality of the subscales. The cutoff values used in previous studies were considered [41,42]. Subsequently, the Automatic Item Selection Procedure (AISP) was used to divide the item set into unidimensional scales [43]. In Phase 4, a confirmatory factor analysis (CFA) was conducted on the polychoric correlation matrix obtained from the second random subsample (n = 500). The reliability of the measures was then assessed using this model, examining internal consistency, reliability of individual indicators, construct reliability and measurement error. Specifically, ordinal alpha [44] and McDonald’s omega [45,46] were calculated. Subsequently, measurement invariance of the established EDS-E factor structure across gender was evaluated using multi-group confirmatory factor analysis (MGCFA). A sequence of nested models testing configural, metric, scalar and strict invariance was evaluated. Invariance between models was supported if the change in CFI (ΔCFI) was ≤ 0.010 and the change in RMSEA (ΔRMSEA) was ≤ 0.015 [47,48].

All models were estimated using diagonally weighted least squares on the polychoric correlation matrix using R version 4.4.1 (14 June 2024) [49] and the lavaan package [50]. The Diagonally Weighted Least Squares (DWLS) estimator was selected because the EDS-E items use an ordinal Likert-type scale response format. DWLS is considered more robust than Maximum Likelihood (ML) for analyzing ordinal data, particularly when assumptions of multivariate normality might not be fully met, as is common with scale data [51].

## 3. Results

### 3.1. Sample Adequacy Index, Multivariate Outliers, and Guttman Errors

An adequate sample adequacy index was obtained. The values obtained were KMO = 0.89, and Bartlett’s sphericity test χ^2^(528) = 307.79; *p* < 0.001. All nine items obtained MSA values close to or above 0.9 (DISCRI1= 0.89, DISCRI2 = 0.88, DISCRI3 0.90, DISCRI4 0.92, DISCRI5 0.85, DISCRI6 0.90, DISCRI7 = 0.90, DISCRI8 = 0.90, DISCRI9 0.90). Results of multivariate outlier analysis show that none of the D^2^ distance values were significant at the α = 0.001 significance level [52], with the maximum D^2^ value being 23.97. The number of Guttman errors was calculated to identify atypical response patterns with the identification of eight cases with atypical response patterns. These results can be observed in detail in the Supplementary Materials (Figures S1 and S2).

### 3.2. Unidimensionality Analysis

Regarding the homogeneity of the items, the coefficients (H) are examined for the set of items (for each item, item pair and the overall scale). The H scalability values of all items are shown in Table 1. All items exceeded the critical value 3. The overall scalability coefficient obtained for the nine items was H = 0.376 (SE = 0.014). The scalability of the item pairs was above 0.3 for all pairs except for item pair 7–5 (Hij = 0.264, SE = 0.041), for item pair 8–6 (Hij = 0.279, SE = 0.035) and for item pair 9–7 (Hij = 0.282, SE = 0.037). Thus, no indications of multidimensionality were identified, and the items are scalable to H ≥ 0.30, indicating medium accuracy [42].

The Automated Item Selection Procedure (AISP) was then carried out at increasing threshold levels of homogeneity to examine dimensionality. If all items appear as belonging to dimension number 1, it is an indicator that the scale is unidimensional at that threshold of homogeneity (indicated in the column headings from 0.1 to 0.5). Table 2 shows the results of the automated item selection procedure; it can be seen that the set of three items can be considered unidimensional with a homogeneity threshold H ≥ 0.3. Furthermore, all items showed local independence (i.e., they are only related through the latent variable).

Regarding the monotonicity assumption, Table S2 (Supplementary Materials) shows the results of the analysis indicating that there are no significant violations (#zsig) of monotonicity for any of the items. In other words, all items appear to discriminate well between participants with high levels of discrimination and those with lower levels. Subsequently, the presence of possible floor and ceiling effects was identified. A floor effect is observed with 14.7% of the participants responding the lowest option in all items. In the Supplementary Materials, the results of this analysis can be seen in Figure S3.

Table 3 shows the expected and observed correlations between each item and the total score, excluding the item itself [53]. A lower observed correlation than expected indicates that the item may not fit the dimension, while a higher observed correlation suggests overfitting or redundancy.

### 3.3. Confirmatory Factor Analysis

Model fit indices indicated excellent fit for the proposed single-factor model (χ^2^(27) = 48.144, RMSEA = 0.031, CFI = 0.993, TLI = 0.991, SRMR = 0.046). Regarding the evidence of supporting the scale’s internal structure, as shown in Table 4, (a) the factor loadings of all indicators were significant; (b) all of them were above 0.4. This indicates that the items strongly and effectively represent the underlying construct of everyday discrimination. In the Supplementary Materials, it is possible to observe the monotonically increasing step response function (ISRF) and the characteristic curves of each item (Figures S4 and S5), and Table S4 shows the values of the principal component analysis of the residuals.

A series of nested models were tested to assess measurement invariance across gender groups. Initially, a configural invariance model was specified, allowing for free estimation of factor loadings and intercepts. Subsequently, more restrictive models were tested: metric invariance (equal factor loadings), scalar invariance (equal factor loadings and intercepts) and strict invariance (equal factor loadings, intercepts and error variances). To evaluate the significance of differences in model fit between these nested models, chi-square difference tests were conducted, and the recommendations of Chen [47] and Cheung and Rensvold [48] were applied, considering decreases in CFI and TLI of less than 0.01 and increases in RMSEA of less than 0.015 as negligible changes in model fit.

The initial model, representing configural invariance, examines the fundamental structure of the measurement model. It posits an identical pattern of factor loadings across the groups under comparison; that is, the same items load onto the same factors in both groups. In this instance, the baseline model tests the hypothesis that the overall pattern of factor loadings remains constant across both groups.

The fit indices indicated adequate model fit for the configural invariance model (χ^2^(54) = 60.56, *p* = 0.251; CFI = 0.998; RMSEA = 0.017, 95% CI [0–0.036], *p*(RMSEA < 0.05) = 0.999), supporting the assumption that these items represent the same underlying construct for both groups. It can be concluded that the pattern of item factor loadings is similar across the two groups. Table 5 presents the goodness of fit information for each model (χ^2^(df), CFI, TLI, RMSEA) and the model comparisons (ΔTLI, ΔCFI, ΔRMSEA).

Moreover, the chi-square difference test was not significant, χ^2^(8) = 12.573, *p* = 0.127, indicating that the factor loadings were equivalent across genders. The evidence of metric invariance suggests that the items contribute consistently to the latent variable across groups. This allows for comparisons of latent variable variances and covariances between groups and warrants further investigation of scalar invariance.

To assess scalar invariance, the model with invariant factor loadings (metric invariance) was compared to a model with invariant intercepts (scalar invariance). The latter model imposes the additional constraint that the intercepts of each indicator are equal across groups. Scalar invariance allows for meaningful comparisons of latent means between groups. As shown in Table 5, the scalar invariance model did not significantly worsen the fit compared to the metric invariance model. The chi-square difference test was not significant, χ^2^(8) = 11.27, *p* = 0.187, supporting the assumption of scalar invariance.

Finally, the strict invariance model tests the null hypothesis that the error variances of each item are equivalent across both groups. In this case, group differences in the observed variables can be attributed solely to differences in the underlying latent factors. Therefore, any observed group differences in the manifest variables are a result of differences in the latent factors. As shown in Table 5, this model did not significantly worsen the fit compared to the less restrictive scalar invariance model. The chi-square difference test was not significant (χ^2^(9) = 12.78, *p* = 0.173), supporting the assumption of strict invariance.

### 3.4. Group Differences in Latent Means

After establishing strict measurement invariance across both genders, we compared the latent mean differences between male and female groups. To test for these differences, two models were compared. In the first model, factor loadings, intercepts, error variances and factor means were constrained to be equal across groups. In the second model, only the factor means were allowed to vary. The male group exhibited a latent mean 0.021 lower than the female group. However, as shown in Table 6, no significant differences were found in the level of discrimination between the two groups (χ^2^(1) = 0.608, *p* = 0.436, d = 0.03).

To examine the differential impact of minority ethnic group membership, physical or mental health conditions, disability status and family educational background on perceived discrimination, a multiple-indicator multiple-causes (MIMIC) model [54] was employed. The MIMIC model allowed us to investigate the direct and indirect effects of these group memberships on a latent construct representing experiences of discrimination, while accounting for measurement error in the observed indicators of discrimination.

The results indicate a satisfactory model fit (χ^2^(35) = 39.705, CFI = 0.999, TLI = 0.999, RMSEA = 0.012, SRMR = 0.040). Our findings revealed that physical, mental or disability-related health conditions were significantly associated with increased perceptions of discrimination (β = 0.917, z = 6.96, *p* < 0.01). In contrast, neither ethnic minority status (β = 0.054, z = 0.91, *p* = 0.361) nor age (β = 0.017, z = −1.55, *p* = 0.248) was found to be a significant predictor of discriminatory experiences.

## 4. Discussion

This study examined the psychometric properties of the Everyday Discrimination Scale (EDS) in a Spanish sample of adolescents. The EDS is a nine-item self-report measure that assesses experiences of everyday discrimination. The sample consisted of 1000 adolescents (50.7% female) aged 12–16 years. The random sampling was stratified in terms of age, sex and territorial distribution (sampling error 3.1% with a confidence level of 95.5% for an infinite universe and under the assumption of maximum indeterminacy).

First, confirmatory factor analysis and Mokken scale analysis supported the unidimensionality of the scale. Most previous studies have found that the EDS can be effectively modeled as a unidimensional scale, meaning that all items load on a single factor representing overall perceptions of discrimination [31]. However, the study conducted with Portuguese adolescents [32] revealed a two-factor structure (Unfair Treatment and Personal Rejection) with a hierarchical model in which these two factors are subordinated to a second-order latent factor, Perception of Discrimination. Our results do not support the existence of such a two-factor structure, being coincident with the unidimensional structure found in the study with Chilean adolescents [34]. In this sense, it would be interesting for future studies to test the EDS on samples with different languages to study the possible explanation for the different factorial structures found. It is important to highlight the results of the qualitative work of Harnois [55], in which it was found that people may interpret the EDS questions in very different ways: some interpret the scale as a question about negative interactions; others see it from the point of view of social inequalities; and others from the specific point of view of racism. Racial/ethnic and gender status may structure the interpretation of the questions [55], which could be a possible explanation for the discrepancies in the number of factors found on the scale in different samples.

Specifically, the results in our sample of Spanish adolescents indicated that the EDS is invariant across gender groups, suggesting that it measures discrimination similarly in males and females. This result is congruent with the reference work of Bastos and Harnois [28], in which multigroup confirmatory factor analyses were conducted to compare the configurational, metric and scalar structures of the EDS in its original English version. The invariance across gender groups allows us to establish the relationship between discrimination and health-related variables [28]. The results of our study indicate that physical, mental or disability-related health conditions were significantly associated with increased perceptions of discrimination. These data are congruent with previous studies indicating that discrimination in adolescence is related to higher rates of anxiety and depression [8], higher levels of stress and psychological maladjustment [7] or, for example, with a higher risk of suffering from a substance addiction problem or attention deficit hyperactivity disorder [9].

If we take as a reference the American Psychological Association (APA) [56] definition of discrimination, namely “unfair or prejudicial treatment of individuals and groups based on characteristics such as race, gender, age or sexual orientation”, it becomes of great interest to analyze the results in terms of group identity. There is evidence that discrimination among adolescents in Spain is multifaceted and affects various groups differently depending on their ethnicity [13] or gender [14], among others. In this regard, no significant differences were found in perceived discrimination between males and females. While some studies suggest that there are no significant gender differences among adolescents [29] on overall EDS scores, others highlight that boys, especially those from minority backgrounds, may perceive higher levels of discrimination [57,58]. Future studies could explore how sexism affects such discrimination, as in the study with Spanish adolescents by Gil Bermejo et al. [14] it was a very important element in explaining discrimination towards females. Contextual factors and the specific environments in which discrimination occurs also play a crucial role in shaping these experiences [59]. However, in our study, neither ethnic minority status nor age was found to be a significant predictor of discriminatory experiences. It would be of interest for future studies to focus on studying the phenomenon with specific minority samples of Spanish adolescents in order to understand all the nuances essential for developing interventions aimed at addressing discrimination among adolescents

The findings provide evidence that the EDS is a reliable and valid measure of everyday discrimination in Spanish adolescents. The first and clearest implication of this work is that it provides assurance that the EDS is an appropriate instrument for measuring the perception of discrimination in Spanish adolescents. Future research will be able to rely on psychometric guarantees when using the EDS with Spanish adolescents. This is very important because the EDS allows us to measure perceptions of unfair treatment derived from everyday discrimination, which is something different from general interpersonal aggression, highlighting the importance and usefulness of the present study. In this way, the instrument can be used to detect risk situations and to test interventions against a control group, to help identify the most effective strategies to reduce discrimination [60]. On the other hand, the EDS should be used with intersectionality in mind, as marginalized people often experience discrimination on multiple grounds [55]. This approach can help capture the full scope of discrimination experiences and provide more accurate data for interventions. With evidence that the EDS has adequate psychometric properties in the study population, future research in this regard will have greater assurance of quality.

The study has some limitations that should be acknowledged. The cross-sectional nature of this study limits the ability to draw causal inferences about the relationship between discrimination and its potential consequences. The sample was drawn from a panel of families with internet access, potentially excluding adolescents from lower socioeconomic backgrounds who may have different experiences of discrimination. In addition, the reliance on self-reported measures of discrimination, while common in this field of research, introduces potential biases. Adolescents’ perceptions and interpretations of discrimination might be influenced by individual factors, social desirability, or recall difficulties. This study partially addressed this by including parent reports of their children’s health conditions, but other variables were solely adolescent-reported. Furthermore, validity tests based on relationships with other variables have not been carried out. On the other hand, the EDS captures general experiences of everyday discrimination without specifically targeting the reasons for discrimination. While participants could indicate the reasons they perceived were behind the discriminatory actions, this study did not deeply analyze these or examine whether the psychometric properties of the EDS hold across different types of discrimination. A floor effect was identified, with 14.7% of participants reporting the lowest possible level of discrimination across all items. This suggests the EDS might not be sensitive enough to capture the experiences of adolescents who encounter very low levels of discrimination, potentially underestimating the prevalence of discrimination in the sample. In addition, it should be noted that these floor effects have been previously reported by other authors [61], particularly in populations that experience low levels of discrimination. These data highlight the need for careful consideration and appropriate statistical methods to ensure accurate measurement and analysis of discrimination experiences. Researchers should be aware of these limitations and adopt strategies to address them effectively in order to adequately measure discrimination.

Future research may benefit from understanding the psychometric properties of the EDS-E questionnaire in order to deepen knowledge about discrimination among adolescents. Studies that highlight experiences of discrimination and relate them to various causes and sociodemographic groups will be essential for identifying key vulnerability factors and for implementing subsequent interventions. In addition, upcoming research should employ longitudinal designs to examine the temporal relationship between discrimination and various outcomes. Examining the psychometric properties and prevalence of discrimination in more diverse samples, including adolescents from different countries, socioeconomic backgrounds, ethnicities and with varying disabilities, is crucial. Combining quantitative approaches like the EDS with qualitative methods (e.g., interviews, focus groups) could provide richer insights into the lived experiences of discrimination among adolescents. Qualitative data can illuminate the nuances of how discrimination is perceived, coped with, and the specific contexts in which it occurs. A critical next step is to develop and rigorously evaluate interventions aimed at reducing discrimination and its impact on adolescents. These interventions could target individual coping strategies, school climate or broader societal attitudes. Also, the intersectionality of different forms of discrimination should be considered. Examining how multiple marginalized identities (e.g., being a girl from an ethnic minority group with a disability) interact to shape experiences of discrimination is essential for developing tailored interventions. By addressing these limitations and pursuing these research directions, the field can advance our understanding of everyday discrimination and its consequences for adolescents, ultimately contributing to the creation of a culture of equality.

## Figures and Tables

**Table 1:** Homogeneity coefficients.

Item	Item H	se
DISCRI1	0.417	(0.017)
DISCRI2	0.435	(0.017)
DISCRI3	0.374	(0.021)
DISCRI4	0.382	(0.018)
DISCRI5	0.340	(0.023)
DISCRI6	0.374	(0.019)
DISCRI7	0.353	(0.021)
DISCRI8	0.346	(0.020)
DISCRI9	0.348	(0.020)

**Table 2:** MSA—AISP for increasing H thresholds (t).

Item	t = 0.10	t = 0.15	t = 0.20	t = 0.30	t = 0.35	t = 0.40	t = 0.45	t = 0.50
DISCRI1	1	1	1	1	1	1	1	1
DISCRI2	1	1	1	1	1	1	1	1
DISCRI3	1	1	1	1	1	1	1	0
DISCRI4	1	1	1	1	1	1	0	0
DISCRI5	1	1	1	1	0	0	0	0
DISCRI6	1	1	1	1	1	1	0	0
DISCRI7	1	1	1	1	1	0	0	0
DISCRI8	1	1	1	1	2	2	0	0
DISCRI9	1	1	1	1	2	2	0	0

**Table 3:** Expected and observed correlations items—total score.

Item	Observed Value	Model Expected Value	Absolute Difference	Adjusted *p*-Value (BH)	Significance Level
DISCRI1	0.54	0.51	0.03	0.242	
DISCRI2	0.58	0.50	0.08	0.001	**
DISCRI3	0.56	0.53	0.03	0.483	
DISCRI4	0.51	0.50	0.01	0.610	
DISCRI5	0.49	0.54	0.05	0.291	
DISCRI6	0.54	0.52	0.02	0.534	
DISCRI7	0.35	0.46	0.11	0.000	***
DISCRI8	0.43	0.50	0.07	0.015	*
DISCRI9	0.51	0.54	0.03	0.345	

Note: * *p* < 0.05; ** *p* < 0.01; *** *p* < 0.001

**Table 4:** Estimates of the one-dimensional solution.

Item	B	SE	Z	Beta	R^2^
1. You are treated with less courtesy or politeness than other people.	0.725	0.026	28.072	0.682	0.465
2. You are treated with less respect than other people.	0.807	0.028	28.666	0.748	0.559
3. You are treated worse than other people in a restaurant or a shop.	0.455	0.021	22.023	0.532	0.284
4. People act as if they think you are not intelligent.	0.765	0.029	26.456	0.659	0.434
5. People act as if they are afraid of you.	0.333	0.019	17.161	0.426	0.182
6. People act as if they think you are dishonest or untrustworthy.	0.580	0.024	24.521	0.630	0.397
7. People act as if they are better than you,	0.860	0.031	28.040	0.633	0.400
8. They call you names or insult you.	0.601	0.025	23.835	0.582	0.339
9. You are threatened or assaulted.	0.437	0.019	23.546	0.591	0.349

Note: All *p*-values were statistically significant at *p* < 0.001.

**Table 5:** Measurement invariance across gender.

Invariance Model	χ^2^			RMSEA		ΔRMSEA	CFI	ΔCFI	TLI	ΔTLI
	Value	df	*p*	Value	90% CI					
Configural	60.56	54	0.251	0.017	[0.0–0.036]	-	0.998	-	0.997	-
Metric	73.13	62	0.158	0.021	[0.0–0.037]	0.004	0.996	−0.002	0.996	−0.001
Scalar	84.40	70	0.116	0.022	[0.0–0.038]	0.001	0.995	−0.001	0.995	−0.001
Strict	97.18	79	0.081	0.023	[0.0–0.038]	0.001	0.994	−0.001	0.995	0

Note: RMSEA = root mean square error of approximation; CFI = comparative fit index; TLI = Tucker–Lewis index; df = degrees of freedom; CI = confidence interval; Δ (CFI, TLI, RMSEA, SRMR) = changes in fit with respect to the previous least restrictive model. Configural = (for identification purposes) one marker variable per factor fixed to 1, unique variances of marker variables fixed as 1; unique variance of first group fixed as 1, factor means of first group fixed as 0; Metric = loadings constrained to be equal across group; Scalar = factor loadings and thresholds constrained to be equal across group; Strict = all unique variances of all groups fixed to 1.

**Table 6:** Latent means differences.

Factor	χ^2^			RMSEA			CFI	ΔCFI	est	z	*p*	Effect Size
	Value	df	*p*	Value	90% CI	*p* < 0.05						*d*
Equal means	101.41	81	0.062	0.025	[0.0–0.038]	0.999	0.993	-	-	-	-	-
Unequal means	100.81	80	0.058	0.025	[0.0–0.039]	0.999	0.993	0	−0.021	−0.78	0.435	0.03

Note: CFI = comparative fit index; df = degrees of freedom; RMSEA = root mean square error of approximation; TLI = Tucker–Lewis index; CI = confidence interval.

## Data Availability

The data presented in this study are available upon request from the corresponding author due to privacy reasons.

## Supplementary Materials

The following supporting information can be downloaded at: https://www.mdpi.com/article/10.3390/jcm14092887/s1, Table S1: Sociodemographic characteristics of the sample; Table S2: MSA—Monotonicity Analysis [42]; Table S3: Principal component analysis of the residuals; Figure S1: Q-Q Plot of Mahalanobis D2 vs. Quantiles of χ2; Figure S2: Distribución de Errores de Guttman; Figure S3: Floor/ceiling effects; Figure S4: Step Response Function (ISRF); Figure S5: Item Characteristics Curves.
