# Supplementary material for: Spanish Version of the Everyday Discrimination Scale (EDS-E): Factorial Structure and Scale Invariance in Spanish Adolescents
Source: J Clin Med. 2025 Apr 22;14(9):2887. doi: 10.3390/jcm14092887 (PMC12072784; doi:10.3390/jcm14092887)
Supplement: Supplementary file 1 [file jcm-14-02887-s001.zip › jcm-3566613-supplementary.pdf]

Supplemental material

Table S1

Sociodemographic characteristics of the sample.

Variable	N	%
Gender		
Male	493	49.3
Female	500	50
None of the above	7	0.7
Age		
12	199	19.9
13	199	19.9
14	203	20.3
15	200	20
16	199	19.9
City size		
0-10.000	231	23.1
10.001-50.000	255	25.5
50.001-200.000	226	22.6
Más de 200.000	288	28.8
Monthly family income		
Utntil 2160	419	41.9
Between 216-4000	474	47.4
More than 4000	107	10.7
Family studies		
No studies	3	0.3
Primary	39	3.9
Basic	382	38.2
University	445	44.5
Master / PhD	131	13.1
Ethnicity		
European	928	92.8
East Europe	38	3.8
Gipsy	3	0.3
Asian	6	0.6
Latin-American	17	1.7
Afro-descendant or black	2	0.2
Arabic or Maghrevi	4	0.4
Prefers not to say	15	1.5
Educative Center		
Public	695	69.5
Concerted	256	25.6
Privat	49	4.9

Mental disorder, illness or disability		
Yes	73	7.3
No	927	92.7

Table S2

MSA — Monotonicity Analysis (see: Stochl et al., 2012)

Item	Item H	#ac	#vi	#vi/#ac	maxvi	sum	sum/#ac	zmax	#zsig	crit
DISCRI1	0.42	140	0	0	0	0	0	0	0	0
DISCRI2	0.43	140	1	0.01	0.05	0.05	0.0003	0.67	0	5
DISCRI3	0.37	140	0	0	0	0	0	0	0	0
DISCRI4	0.38	140	0	0	0	0	0	0	0	0
DISCRI5	0.34	126	6	0.05	0.08	0.37	0.003	1.28	0	29
DISCRI6	0.37	140	0	0	0	0	0	0	0	0
DISCRI7	0.35	133	10	0.08	0.08	0.51	0.0038	1.42	0	34
DISCRI8	0.35	140	0	0	0	0	0	0	0	0
DISCRI9	0.35	140	0	0	0	0	0	0	0	0

Note. #ac = Active Comparisons; #vi = Violations; # zsig = Significant violations

Table S3

Principal component analysis of the residuals

Eigenvalues	Proportion of variance
1.44	16.4%
1.32	16%
1.26	13.5%
1.12	12.5%
1.01	12%

Figure S1

Q-Q Plot of Mahalanobis D^2 vs Quantiles of χ^2

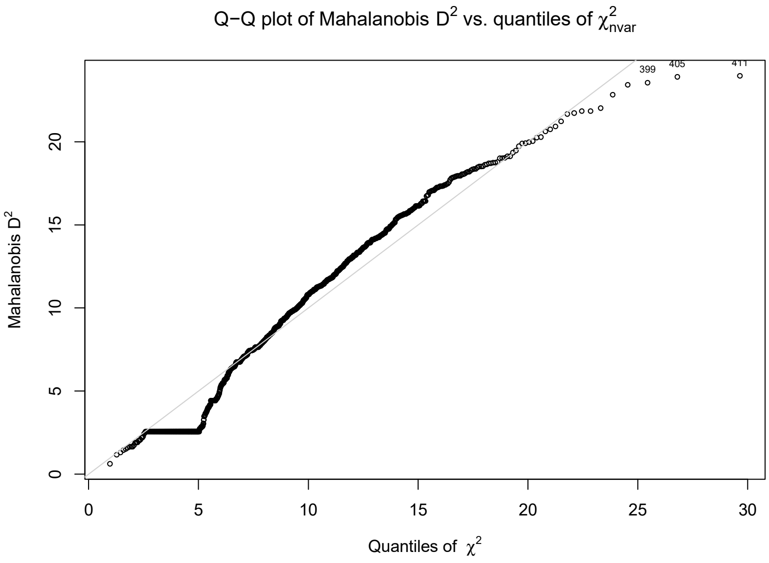

Figure S2

Distribución de Errores de Guttman

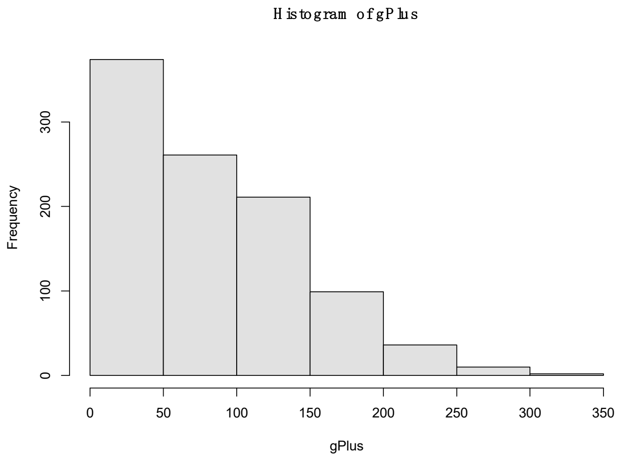

Figure S3

Floor/ceiling effects

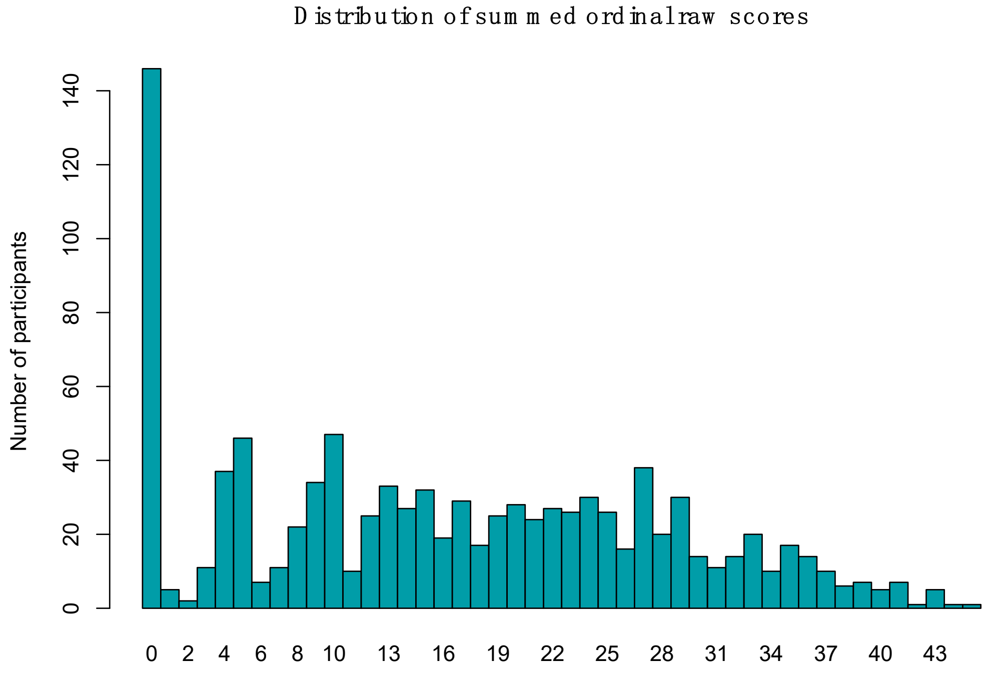

Min score: 14.7% , max score: 0.1%.

Figure S4

Step Response Function (ISRF)

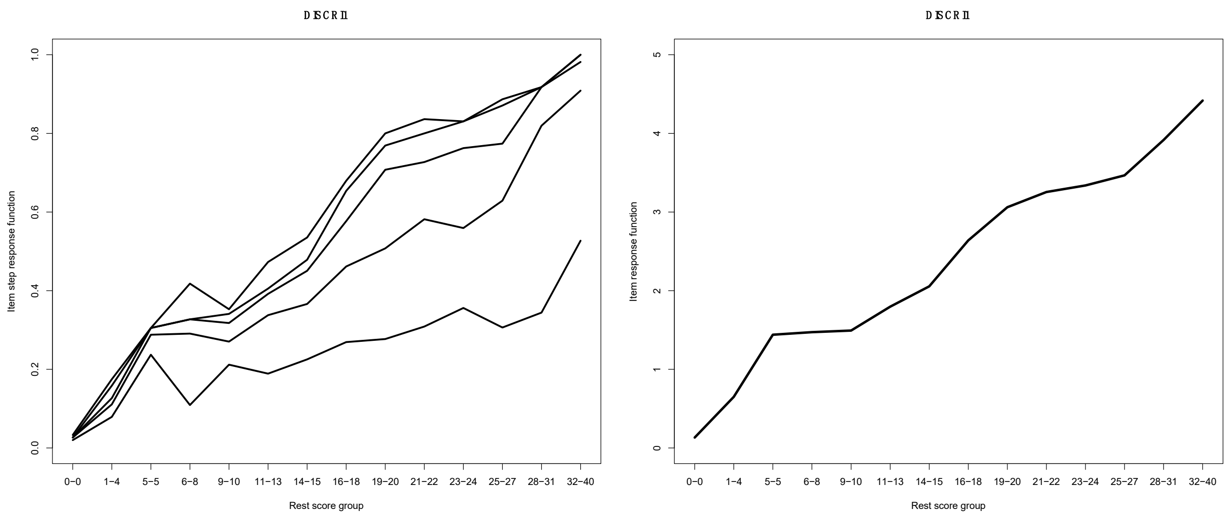

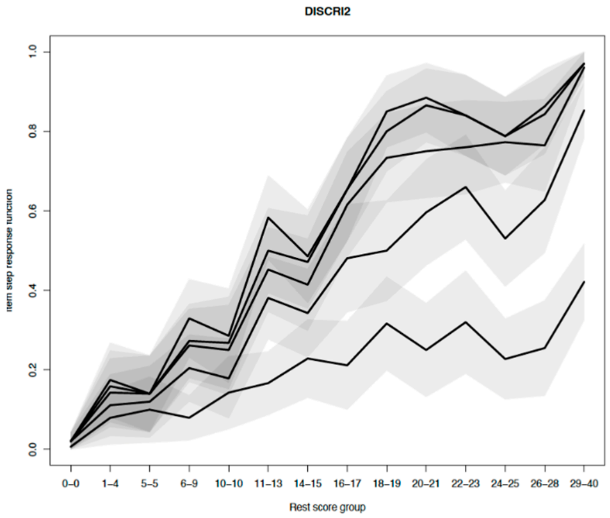

DISCRI2
Item step response function
Rest score group
0–0
1–4
5–5
6–9
10–10
11–13
14–15
16–17
18–19
20–21
22–23
24–25
26–28
29–40

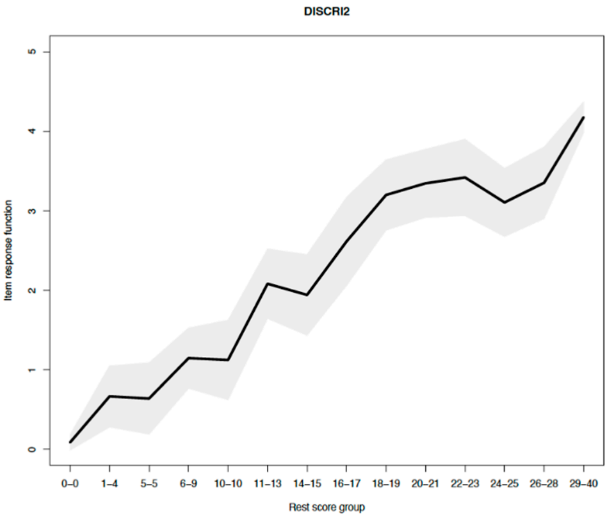

DISCRI2
Item response function
Rest score group
0–0
1–4
5–5
6–9
10–10
11–13
14–15
16–17
18–19
20–21
22–23
24–25
26–28
29–40

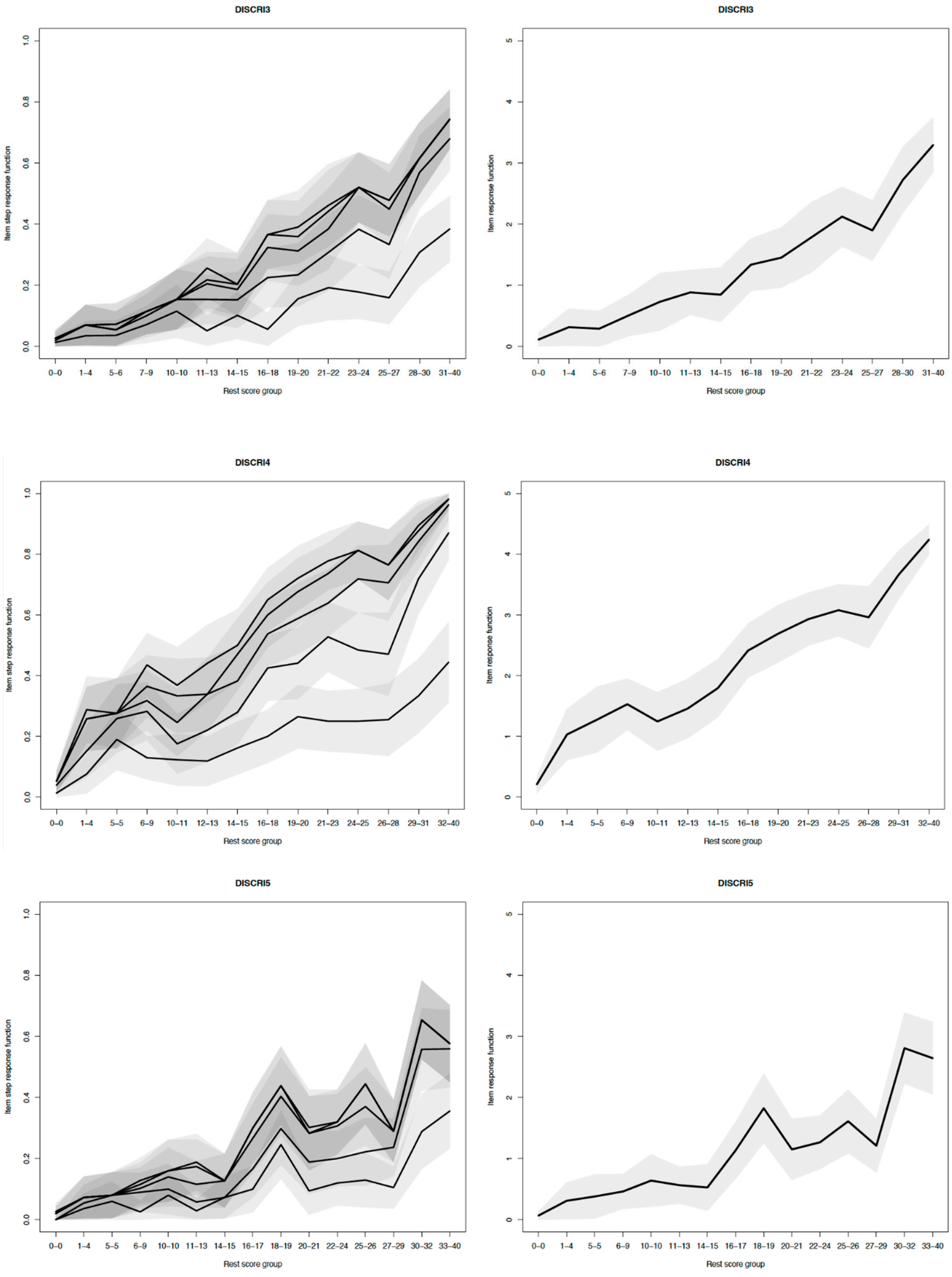
DISCRI3
Item step response function
Item response function
Rest score group
0–0 1–4 5–6 7–9 10–10 11–13 14–15 16–18 19–20 21–22 23–24 25–27 28–30 31–40
DISCRI4
Item step response function
Item response function
Rest score group
0–0 1–4 5–5 6–9 10–11 12–13 14–15 16–18 19–20 21–23 24–25 26–28 29–31 32–40
DISCRI5
Item step response function
Item response function
Rest score group
0–0 1–4 5–5 6–9 10–10 11–13 14–15 16–17 18–19 20–21 22–24 25–26 27–29 30–32 33–40

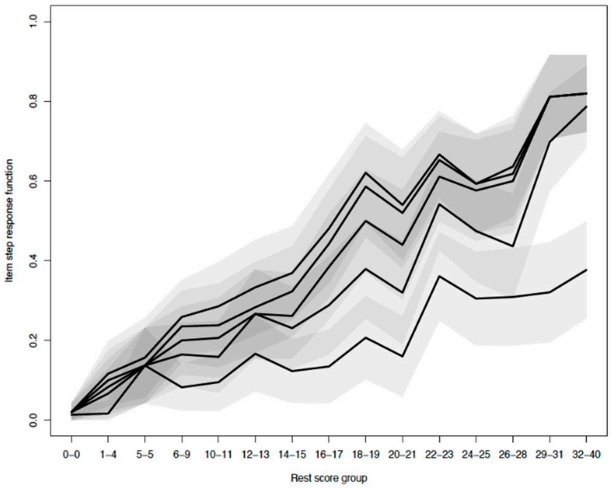

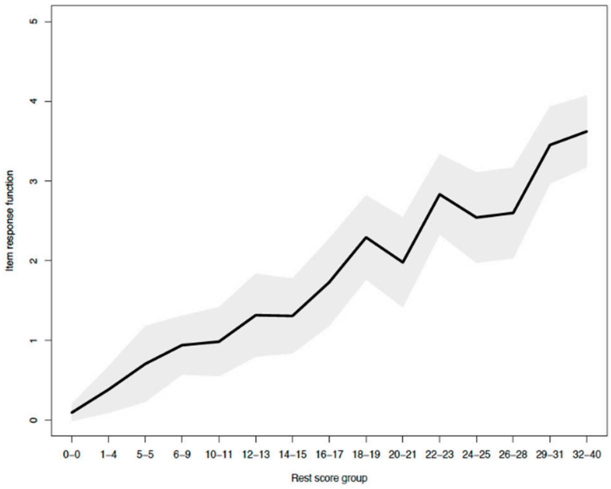

DISCRI7

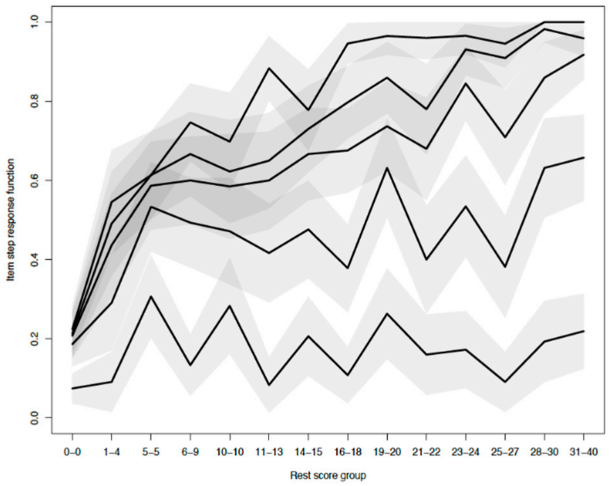

DISCRI7

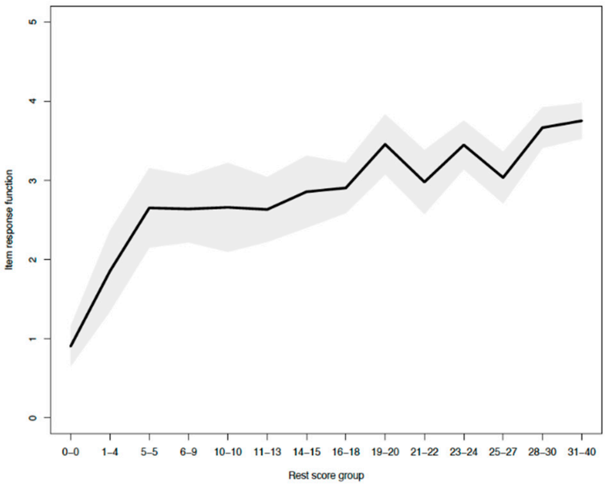

DISCRI8

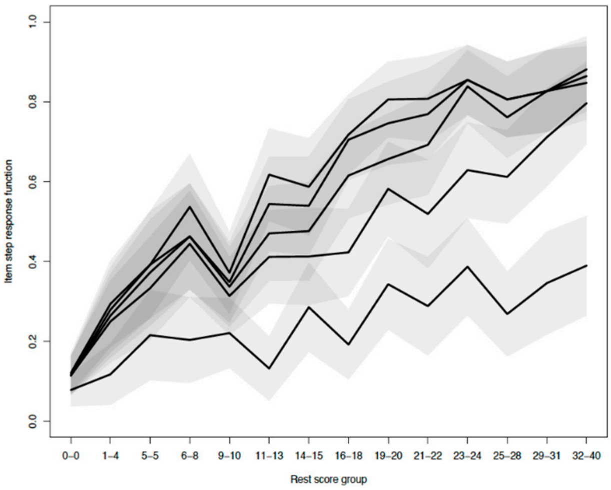

DISCRI8

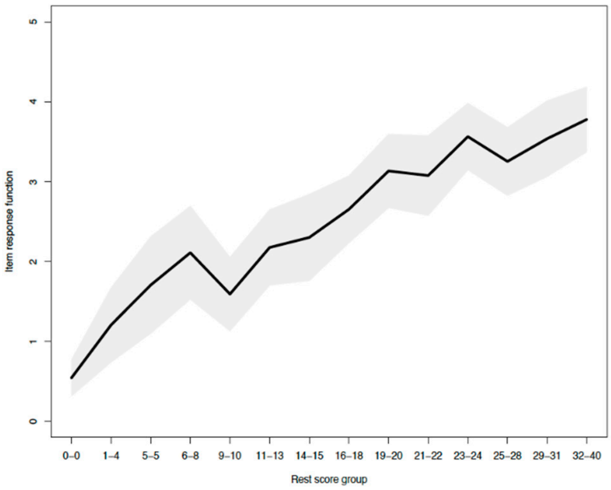

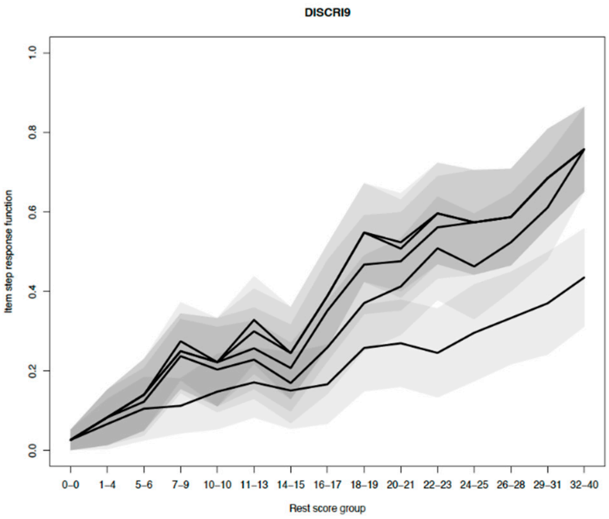
DISCRI9
Item step response function
Rest score group
0–0 1–4 5–6 7–9 10–10 11–13 14–15 16–17 18–19 20–21 22–23 24–25 26–28 29–31 32–40

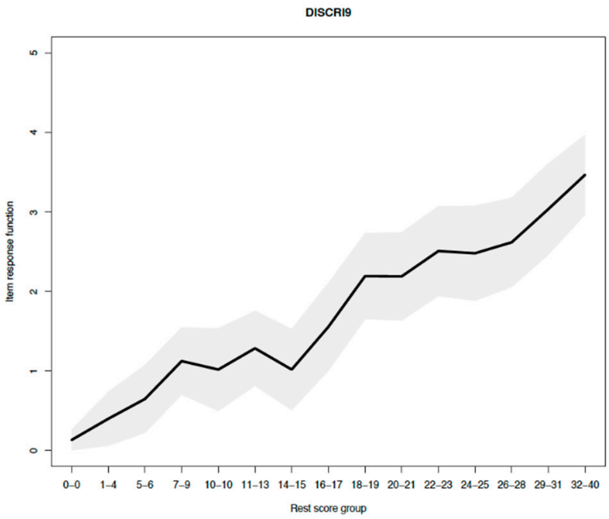
DISCRI9
Item response function
Rest score group
0–0 1–4 5–6 7–9 10–10 11–13 14–15 16–17 18–19 20–21 22–23 24–25 26–28 29–31 32–40

Figure S5

Item Characteristics Curves

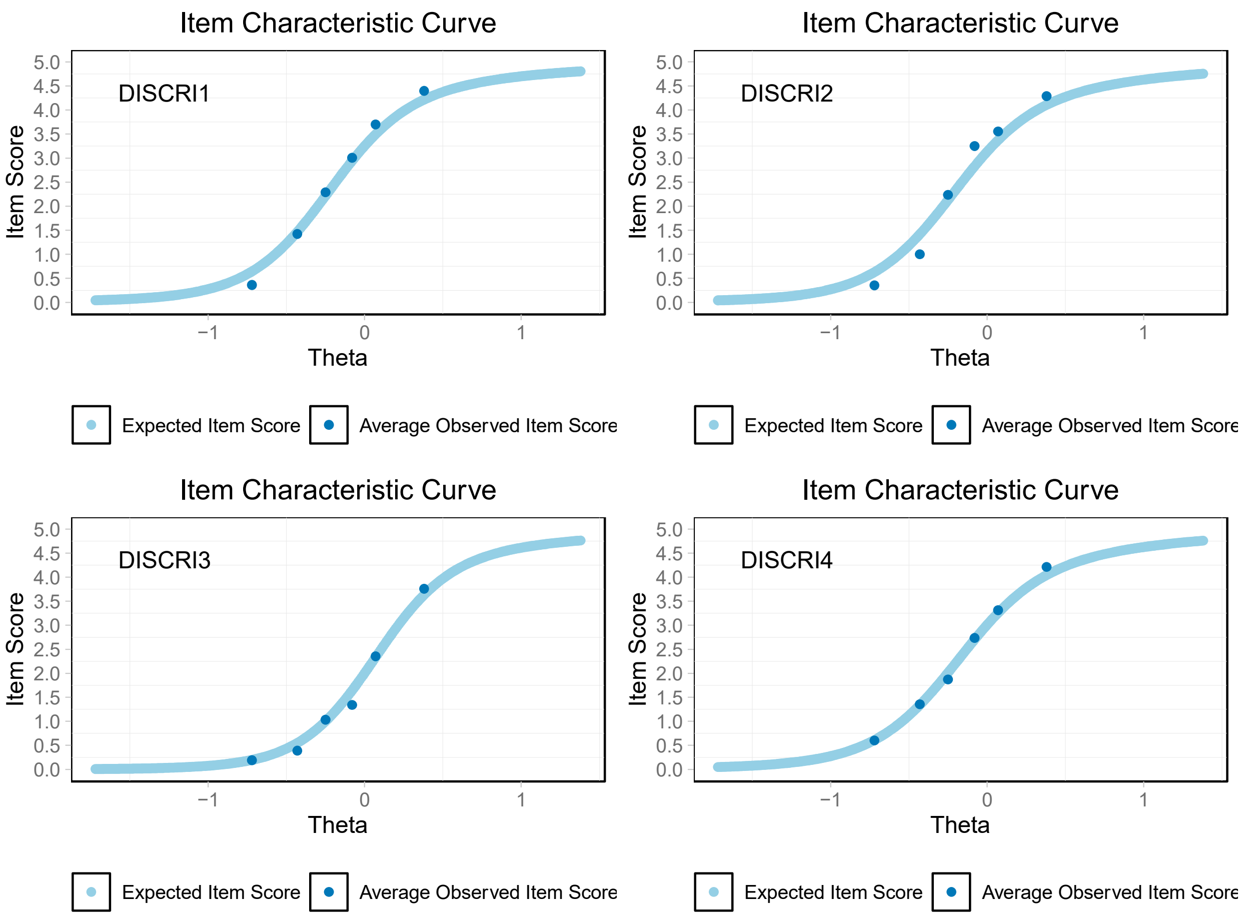

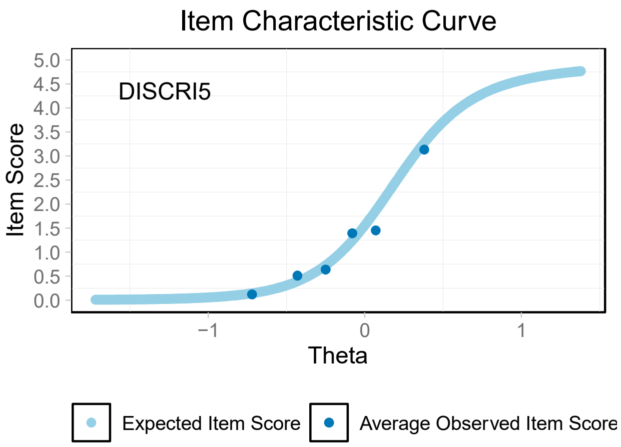
Item Characteristic Curve
DISCRI5
Item Score
5.0
4.5
4.0
3.5
3.0
2.5
2.0
1.5
1.0
0.5
0.0
−1
0
1
Theta
Expected Item Score
Average Observed Item Score

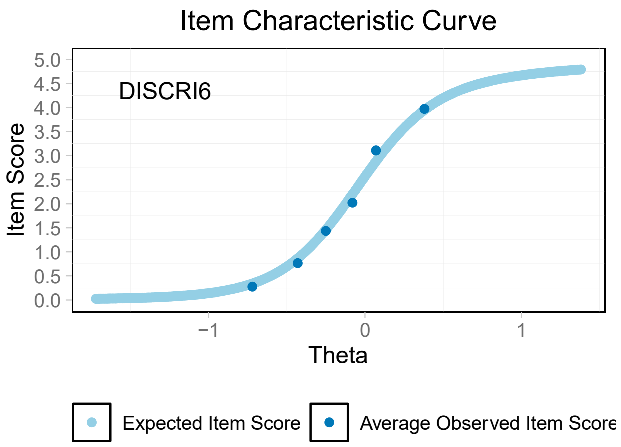
Item Characteristic Curve
DISCRI6
Item Score
5.0
4.5
4.0
3.5
3.0
2.5
2.0
1.5
1.0
0.5
0.0
−1
0
1
Theta
Expected Item Score
Average Observed Item Score

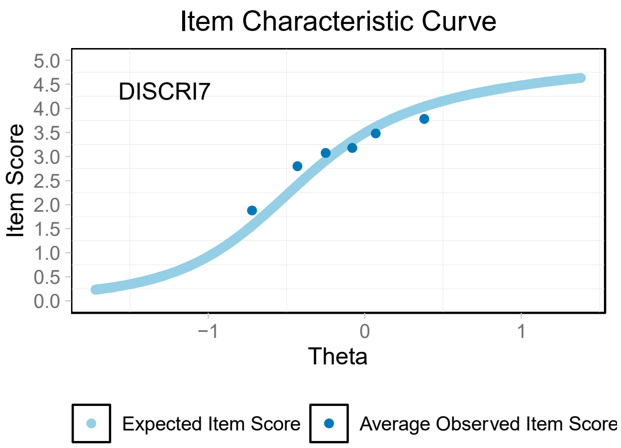
Item Characteristic Curve
DISCRI7
Item Score
5.0
4.5
4.0
3.5
3.0
2.5
2.0
1.5
1.0
0.5
0.0
−1
0
1
Theta
Expected Item Score
Average Observed Item Score

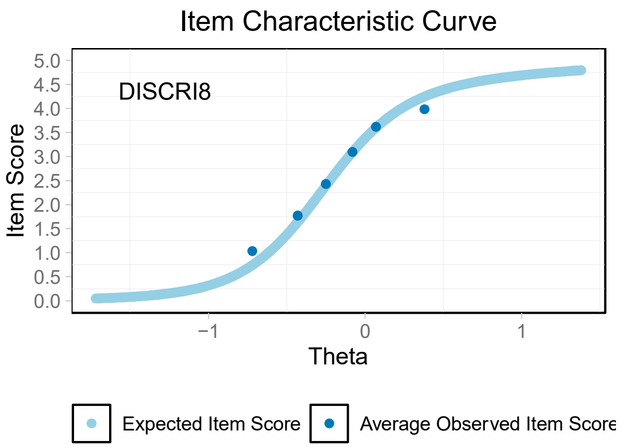
Item Characteristic Curve
DISCRI8
Item Score
5.0
4.5
4.0
3.5
3.0
2.5
2.0
1.5
1.0
0.5
0.0
−1
0
1
Theta
Expected Item Score
Average Observed Item Score

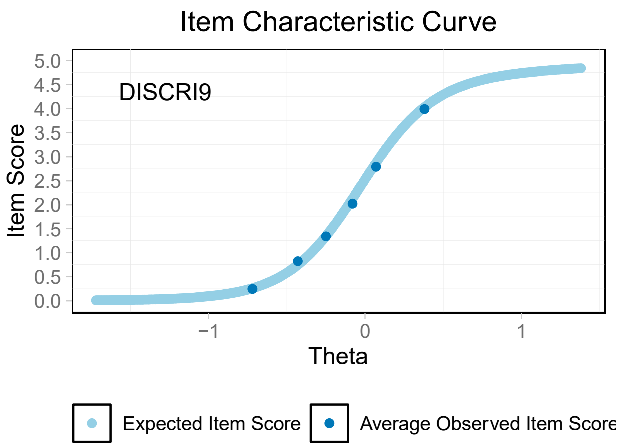
Item Characteristic Curve
DISCRI9
Item Score
5.0
4.5
4.0
3.5
3.0
2.5
2.0
1.5
1.0
0.5
0.0
−1
0
1
Theta
Expected Item Score
Average Observed Item Score
